# Treatment of Lead Poisoning by Iodide of Potassium

**Published:** 1853-11

**Authors:** 


*Treatment of Lead Poisoning by Iodide of Potassium.*—The number of cases of poisoning by lead, received at the New York hospital during the past season, has been quite large. Some of them have been traced to the influence of lead paint as a cause, either in painters or in those who have remained in rooms newly painted; but, in many cases, the mode in which the poison was introduced into the system could not be detected. In cases of constipation, strychnia in doses of one-sixteenth of a grain, three times a day, has been given, and continued until the bowels were opened, or until the specific effects of the drug were produced. Other means have been used to assist its action; and evacuations from the bowels have been produced in some cases by croton oil, and, in other cases, by turpentine enemeta, when croton oil has failed. The strychnia is thought to obviate the tendency to costiveness which follows the use of drastic cathartics. One patient was permanently cured of the constipation by strychnia alone. The blue line about the gums has been recognized in every instance; and in one case there was a patch of deep blue color, of the size of a shilling piece, on the membrane lining the lower lip. All the cases of this disease, after the urgent symptoms are relieved, are now treated with the iodide of potassium, in doses of five grains three times a day, gradually increasing to ten grains, according to the plan proposed by M. Melsens, the results of whose researches have been repeatedly verified by finding lead in the urine after the exhibition of this remedy, when none could be detected in the urine of the same individual before taking it.—*N. Y. Medical Times, October*, 1853.

